# Kinetic and thermodynamic evaluation of antioxidant reactions: factors influencing the radical scavenging properties of phenolic compounds in foods

**DOI:** 10.1002/jsfa.70080

**Published:** 2025-07-24

**Authors:** Moeka Yamauchi, Yukino Kitamura, Chihiro Tada, Riku Kato, Hiroaki Gotoh

**Affiliations:** ^1^ Department of Applied Chemistry Yokohama National University Yokohama Japan

**Keywords:** antioxidants, DPPH, free radical, Trolox equivalent antioxidant capacity, phenolic compounds, antiradical activity

## Abstract

**Background:**

Antioxidants can prevent oxidative stress‐induced aging and diseases, and many antioxidant screening tests have been conducted in the pharmaceutical and food industries. However, the potencies of antioxidants calculated using standard evaluation methods are thermodynamic values at best and ignore the kinetic aspects of their reactions. In this study, 56 compounds with four basic structures (phenol, methoxyphenol, hydroquinone and catechol) were prepared for Trolox equivalent antioxidant capacity (TEAC) assay and reaction tracking over time. The reaction tracking results were evaluated in terms of the stoichiometric number (*n*) after 10 s (*n*_10s_), which reflects the kinetic aspect of the reaction, and *n* after 10 min (*n*_10min_), which reflects the thermodynamic aspect.

**Results:**

Parameter *n*_10min_ was highly correlated with the TEAC obtained using a commercial kit. In addition, *n*_10s_ and *n*_10min_ could roughly classify the reaction patterns of the four basic structures of antioxidants. The relative positions of the hydroxy and methoxy groups in methoxyphenol, hydroquinone, catechol, etc., greatly influenced *n*_10s_ and *n*_10min_.

**Conclusion:**

The radical scavenging ability of antioxidants can be better assessed by conducting evaluations from both thermodynamic and kinetic aspects. This study provides a better understanding of the properties of antioxidants during conventional evaluations using 2,2‐diphenyl‐1‐picrylhydrazyl measurements. © 2025 The Author(s). *Journal of the Science of Food and Agriculture* published by John Wiley & Sons Ltd on behalf of Society of Chemical Industry.

## INTRODUCTION

A portion of inhaled oxygen is converted into reactive oxygen species, which, in excess, damage cells and contribute to disease and aging.^1^, ^2^, ^3^, ^4^ Hence, antioxidants such as polyphenols are often added to the daily diet to improve life expectancy. Antioxidant capacity is commonly assessed by measuring free‐radical scavenging ability.^5^ The 2,2‐diphenyl‐1‐picrylhydrazyl (DPPH) test is a rapid colorimetric method widely used to assess antioxidant ability because it is simple and inexpensive.^6^, ^7^, ^8^

The simple induction of molecular phenols is known to alter their biological activity and antioxidant capacity. For instance, the ferulic acid in vinegar undergoes side‐chain reduction during spontaneous fermentation and a change in structure to dihydroferulic acid, with a corresponding decrease in half maximal inhibitory concentration (IC_50_) of approximately 60–70% (i.e. its radical scavenging capacity (RSC) increases).^9^ Zingerone, which is present in ginger roots and stems, undergoes *o–o* coupling dimerization, which increases its radical scavenging activity (RSA) from approximately 3% to 30–40%.^10^ The *p–p* dimers of propofol and its analogs, which are clinically applied as anesthetics, show 4–10 times higher DPPH RSC than their corresponding monomers.^11^ The number and position of not only phenolic hydroxy groups but also methoxy and carboxylic acid groups exert an important influence on the antioxidant capacity of phenols.^12^

DPPH, a stable free radical, is reduced via the reactions shown in Eqns (1)–(5). Analyses of the reduction of DPPH over the last 20 years suggest that the reaction of phenolic compounds with DPPH occurs via three main mechanisms: direct hydrogen atom transfer^13^ (Eqn 1) from ArOH to DPPH; electron transfer^14^ (Eqns 2 and 3) from ArO^−^ (a low‐concentration phenoxide anion in equilibrium with ArOH) to DPPH; and sequential proton loss electron transfer^15^ (Eqns 4 and 5). The proton‐coupled electron transfer mechanism has recently been proposed to explain the concerted transfer of electrons and protons.^14^, ^16^ These reactions ultimately produce phenoxy radicals and DPPH.

(1) ArOH+DPPH·→ArO·+DPPHH

(2) $$\begin{array}{c} \text{ArOH}+\text{DPPH}\cdot \to \text{ArOH}\cdot^{+}+\mathrm{DPP}H^{-} \end{array}$$

(3) $$\begin{array}{c} \text{ArOH}\cdot^{+}+\mathrm{DPP}H^{-}\to \mathrm{ArO}\cdot +\text{DPPHH} \end{array}$$

(4) $$\begin{array}{c} \text{ArOH}+\text{DPPH}\cdot \to \mathrm{Ar}O^{-}+\text{DPPH}H^{+}\; \end{array}$$

(5) $$\begin{array}{c} \mathrm{Ar}O^{-}+\text{DPPH}H^{+}\to \mathrm{ArO}\cdot +\text{DPPHH}\; \end{array}$$

Depending on its structure, the phenoxy radical follows different reaction pathways; when it reacts with DPPH multiple times, the number of DPPH reductions per molecule of antioxidants increases. This is particularly relevant for compounds with catechol or quinone structures, where a phenoxy radical can sequentially react with two DPPH radicals to form a quinone; the subsequent negative addition of an alcohol solvent finally regenerates the hydroxy group. These reactions lead to strikingly high stoichiometric numbers (*n*) for catechols and hydroquinones.^17^, ^18^ The reaction of DPPH radicals with antioxidants involves multiple competing mechanisms, and the predominant reaction depends on the antioxidant type and experimental conditions; hence, identifying the exact reaction mechanism in a systematic manner is challenging even for a single antioxidant.^15^, ^16^, ^19^, ^20^, ^21^

Common antioxidant metrics like IC_50_ and TEAC cannot be fully explained by indicators such as IP and BDE derived from equations.^1^, ^2^, ^3^, ^4^, ^5^ Research indicates that the properties of antioxidants should be evaluated by distinguishing between antioxidant activity and antioxidant capacity.^5^ Antioxidant activity refers to the initial amount of an antioxidant required to react with a particular oxidant and indicates the kinetics of its inhibitory action. Antioxidant capacity, on the other hand, reflects the *n* of the reaction, that is, the number of oxidant molecules reduced by antioxidant species.^22^ These considerations highlight the need for further analysis of structural features influencing antioxidant behavior.

**Figure 1:**
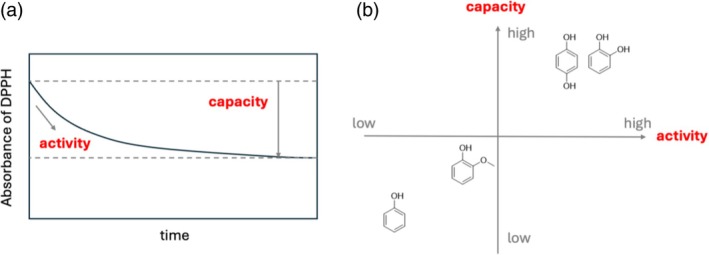
(a) Absorbance of DPPH in a reaction mixture containing antioxidants over time. The initial decrease in absorbance reflects the antioxidant activity, which is a kinetic attribute. The final consumption of DPPH reflects antioxidant capacity, which is a thermodynamic attribute. (b) Capacity and activity of phenols with various structures.

The aim of the study reported here was to better understand the structural factors involved in the RSA and RSC of phenols, which are abundant in food (Fig. 1). To this end, we analyzed the antioxidant properties of 56 low‐molecular‐weight phenolic compounds grouped according to their basic skeleton (methoxyphenol, hydroquinone, catechol and others (including hydroxycinnamic acid and 2,2′‐biphenol)) in terms of their RSA and RSC. In particular, we hypothesize that specific structural features of phenolic compounds – particularly the number and position of hydroxy, methoxy and other substituents – determine their RSA and RSC in DPPH‐based reactions. To test this, we measured the time‐resolved DPPH scavenging behavior of the 56 phenolic compounds using both conventional TEAC assays and direct kinetic tracking. By analyzing the stoichiometric number (*n*) at early (10 s) and late (10 min) time points, we aimed to clarify how structural motifs contribute to both the initial reaction rate and total radical quenching ability. Some of the compounds were synthesized using various organic synthetic reactions, and the DPPH absorbance of all compounds was tracked over time. TEACs were calculated, and the experimental values of RSA and RSC were compared.

## MATERIALS AND METHODS

### Antioxidants

**Figure 2:**
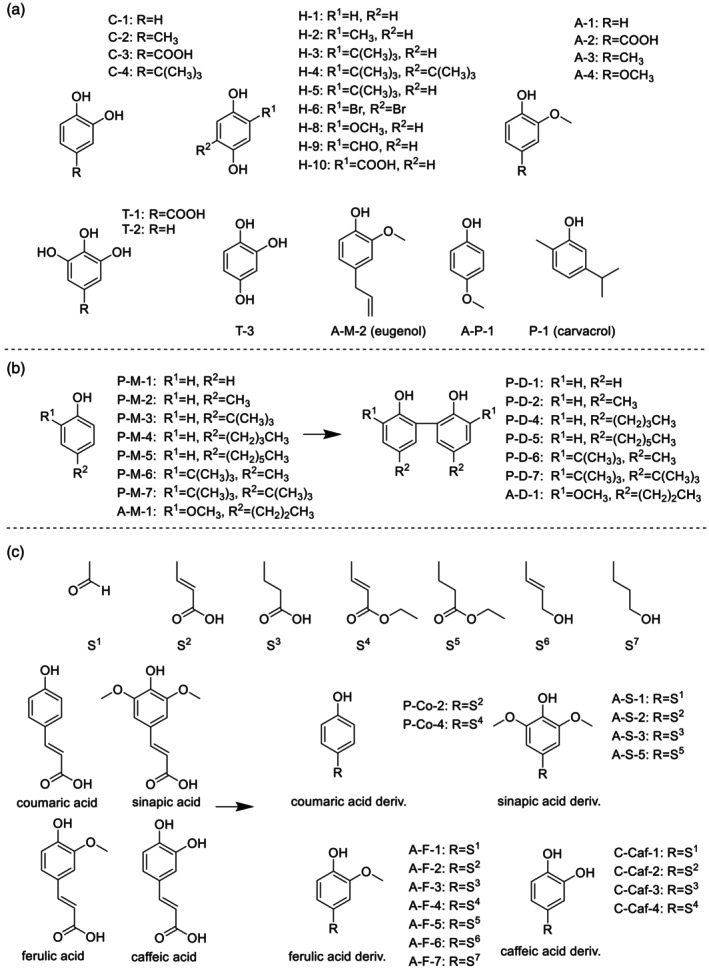
(a) Structures of example commercial compounds. (b) Structures and raw materials of example synthetic compounds. (c) Structures of the derivatives of coumaric acid, ferulic acid, sinapic acid and caffeic acid and their number assignments.

The 56 antioxidants investigated in this study were either commercial or synthetic compounds. The commercial compounds were purchased from Tokyo Kasei Kogyo, FUJIFILM Wako Pure Chemicals or Sigma‐Aldrich, and used as received. The synthetic compounds were synthesized at our laboratory and purified using thin‐layer chromatography. Details of the preparation procedures and ^1^H NMR spectra of the synthesized compounds are provided in the supporting information. The structures of the measured compounds are shown in Fig. 2.

### IC_50_ measurements and TEAC calculations

The TEACs of 47 compounds reported in our previous study were used as data.^23^ Nine other compounds were assayed using DPPH antioxidant assay kits purchased from Dojin Chemical Laboratory Co. Briefly, the DPPH reagent was dissolved in 10 mL of ethanol to prepare a solution. Next, 100 μg mL^−1^ Trolox standard was diluted with ethanol to achieve concentrations of 0, 40, 60, 80 μg mL^−1^, with a final volume of 10 mL. The antioxidants (10 mg) were dissolved in 10 mL of ethanol and diluted to 1, 10, 100 and 1000 μg mL^−1^. The DPPH and antioxidant solutions were prepared on the day of the experiment. The plate reader used was a Skanlt RE for VarioskanFlash 2.4 (Thermo Fisher Scientific). After the optimum concentration ranges were predicted in a preliminary experiment, the IC_50_ and TEAC values of the antioxidant samples were calculated.

A multichannel pipette was used to add 20 μL of the sample antioxidant solution, 80 μL of the assay buffer and 100 μL of the DPPH solution to a 96‐well microplate. Blank 1 was prepared by mixing 20 μL of ethanol, 80 μL of the assay buffer and 100 μL of the DPPH solution, while Blank 2 was prepared by mixing 120 μL of ethanol and 80 μL of the assay buffer. The solution was mixed well by pipetting, and the plate was incubated in the dark at 25 °C for 30 min. The absorbance of the solution at 517 nm was measured using a plate reader, and the RSA of the antioxidant sample was determined using Eqn (6):

(6) $$\begin{array}{c} \mathrm{RSA}\;\left(\%\right)=\frac{A_{\mathrm{CS}}-A_{S}}{A_{\mathrm{CS}}}\times 100 \end{array}$$

where $A_{\mathrm{cs}}=\left(1\right)A_{\text{blank}1}-A_{\text{blank}2}$, $A_{s}=\left(A_{\text{sample}}-\text{sample}A_{\text{blank}2}\right)$, $A_{\text{blank}1}$ is the absorbance of Blank 1, $A_{\text{blank}2}$ is the absorbance of Blank 2 and $A_{\text{sample}}$ is the absorbance of the sample.

A regression line was calculated by plotting the sample concentration on the horizontal axis and RSA on the vertical axis. From the regression line, the optimal concentration range and concentration at which 50% of the RSA of the sample was achieved (IC_50_) were confirmed. The IC_50_ of Trolox was tested in a different cell on the same plate and determined in the same manner. The TEAC of the antioxidant samples was obtained using Eqn (7), and their IC_50_ was interpolated over their linear concentration range. Three measurements were taken for each sample, and the results were averaged.

(7) $$\text{TEAC}\;\left(\text{μmol}\;{\text{μmol}}^{-1}\right)=\frac{{\mathrm{IC}}_{50,\text{Trolox}}\;\left(\text{μmol}\;{\mathrm{mL}}^{-1}\right)}{{\mathrm{IC}}_{50,\text{Sample}}\;\left(\text{μmol}\;{\mathrm{mL}}^{-1}\right)}$$

### Measurement of the reaction over time

The procedures described by Xie *et al*.^24^ were adopted. DPPH was purchased from Tokyo Kasei Kogyo Co., and 1 mol L^−1^ Tris–HCl (pH 7.5) was purchased from Nippon Gene Co. DPPH solution was prepared on the day of the experiment and allowed to stand in the dark for 2 h until the start of the measurement. Antioxidant sample solutions were prepared in ethanol at concentrations of 94.5 and 945 μmol L^−1^. Tris–HCl buffer (1 mol L^−1^, pH 7.5) was diluted with pure water to prepare a 0.1 mol L^−1^ aqueous solution.

A multichannel spectroscopy system, deuterium halide light source and OPwave+, the standard software for spectroscopy measurements (all purchased from Ocean Photonics, Inc.), were used for data acquisition. DPPH solution (1.5 mL) and buffer solution (1.2 mL) were added to a cuvette and purged with Ar gas for 2 min. The cuvette was then loaded into the cuvette holder of the instrument, and the absorbance of the solution was recorded at 517 nm as the initial absorbance. Next, 0.3 mL of 94.5 or 9.45 μmol L^−1^ antioxidant solution was added to the cuvette and rapidly mixed by pipetting to achieve DPPH‐to‐antioxidant concentration ratios ([DPPH]:[antioxidant]) of 1:1 or 10:1, respectively. The absorbance of the solution was recorded at 1 s intervals for 10 min to generate a reaction curve. The solution was continuously mixed using a stirrer during the measurements. Experiments were performed at 25 °C.

### Calculation of *n*

Because the reaction between DPPH and antioxidants is complex, evaluating the reaction rate in a unified manner for a diverse group of compounds is challenging. Parameter *n* is defined as the number of DPPH radicals scavenged per unit of antioxidant, and calculated as the difference in absorbance of the solution before and after the start of the reaction (Eqn 8):

(8) $$n=\frac{∆A}{\varepsilon \;\left(L\;\mathrm{mo}l^{-1}\;cm^{-1}\right)\cdot l\left(\mathrm{cm}\right)\cdot C_{\text{antioxidant}}\;\left(\mathrm{mol}\;L^{-1}\right)}$$

where ∆A is the change in absorbance during the reaction, v is the volume of the DPPH solution, *ε* is the molar absorption coefficient of the solvent, $C_{\text{antioxidant}}$ is the antioxidant concentration and l is the optical path length of the cuvette. In this study, RSA was represented by *n*_10s_, which is the value of *n* obtained 10 s after the start of mixing. Similarly, RSC was represented by *n*_10min_, which is the value of *n* obtained 10 min after the start of the measurement. Measurements were taken three times for each antioxidant and averaged.

## RESULTS AND DISCUSSION

### Evaluation of validity of using antioxidant concentrations to track reaction progress

**Figure 3:**
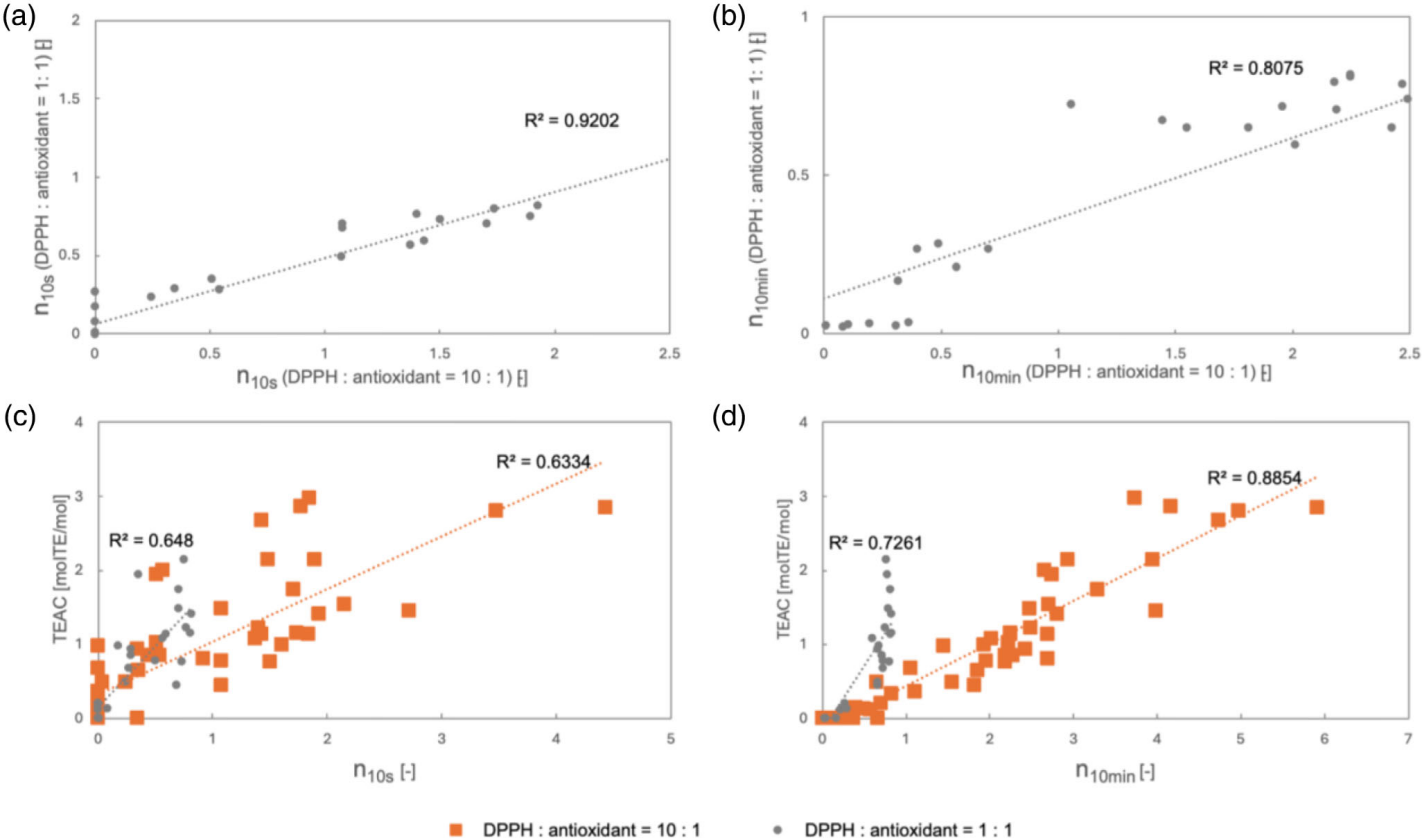
(a) Comparison of stoichiometric numbers (*n*) obtained 10 s after the start of mixing (*n*_10s_) when [DPPH]:[antioxidant] = 10:1 and 1:1. (b) Comparison of *n* obtained 10 min after the start of measurement (*n*_10min_) when [DPPH]:[antioxidant] = 10:1 and 1:1. (c) Comparison of *n*_10s_ and TEAC between [DPPH]:[antioxidant] = 10:1 (orange squares) and 1:1 (gray circles). (d) Comparison of *n*_10min_ and TEAC between [DPPH]:[antioxidant] = 10:1 (orange squares) and 1:1 (gray circles). [DPPH]: 94.5 μmol L^−1^; [antioxidant]: 9.45 or 94.5 μmol L^−1^.

TEAC was calculated by measuring the change in DPPH absorbance over time.^23^ A comparison of *n*_10s_, *n*_10min_ and TEAC was performed in a preliminary study (Fig. 3). A high correlation (*R* ^2^ = 0.88) between *n*_10min_ and TEAC was observed when [DPPH]:[antioxidant] = 10:1. Moreover, *n*_10s_ in the [DPPH]:[antioxidant] = 10:1 condition was highly correlated with *n*_10s_ in the [DPPH]:[antioxidant] = 1:1 condition. Given the findings above, we believe that both the kinetic and thermodynamic aspects of the antioxidation reaction can be evaluated using the [DPPH]:[antioxidant] = 10:1 condition alone. Therefore, our subsequent studies were conducted using [DPPH]:[antioxidant] = 10:1.

### Results of reaction tracking over time

By conducting reaction tracking over time, we obtained some interesting results that supported the findings of previous research. The RSA of zingerone toward DPPH radicals has been reported to increase from approximately 3% to 30–40% by *o–o* coupling dimerization.^10^ In this study, a comparison of the monomers P‐M‐1–7 and A‐M‐1 with the dimers P‐D‐1–7 and A‐D‐1 before and after *o–o* coupling showed a general increase in *n*_10min_ except for A‐M‐1 and P‐D‐6, the reaction rates of which were too slow to compare using *n*_10s_. P‐M‐6 and P‐D‐6, which are comparable, showed an increase in *n*_10s_. The reduction of ferulic acid to dihydroferulic acid through natural fermentation in black vinegar decreases its IC_50_ and increases its antioxidant capacity.^9^ Examination of *n*_10min_ of ferulic acid (A‐F‐2) and its reduced form, dihydroferulic acid (A‐F‐3), revealed a higher *n*_10min_ for A‐F‐2, thus confirming an increase in antioxidant capacity following reduction. However, in terms of *n*_10s_, A‐F‐2 > A‐F‐3. This result indicates the importance of taking measurements over time because the trend of *n* can change as the reaction progresses. Details of the measurement results and other data are provided in the supporting information (Data S1, Data S2 (Figure S1)).

#### Structural classification

**Figure 4:**
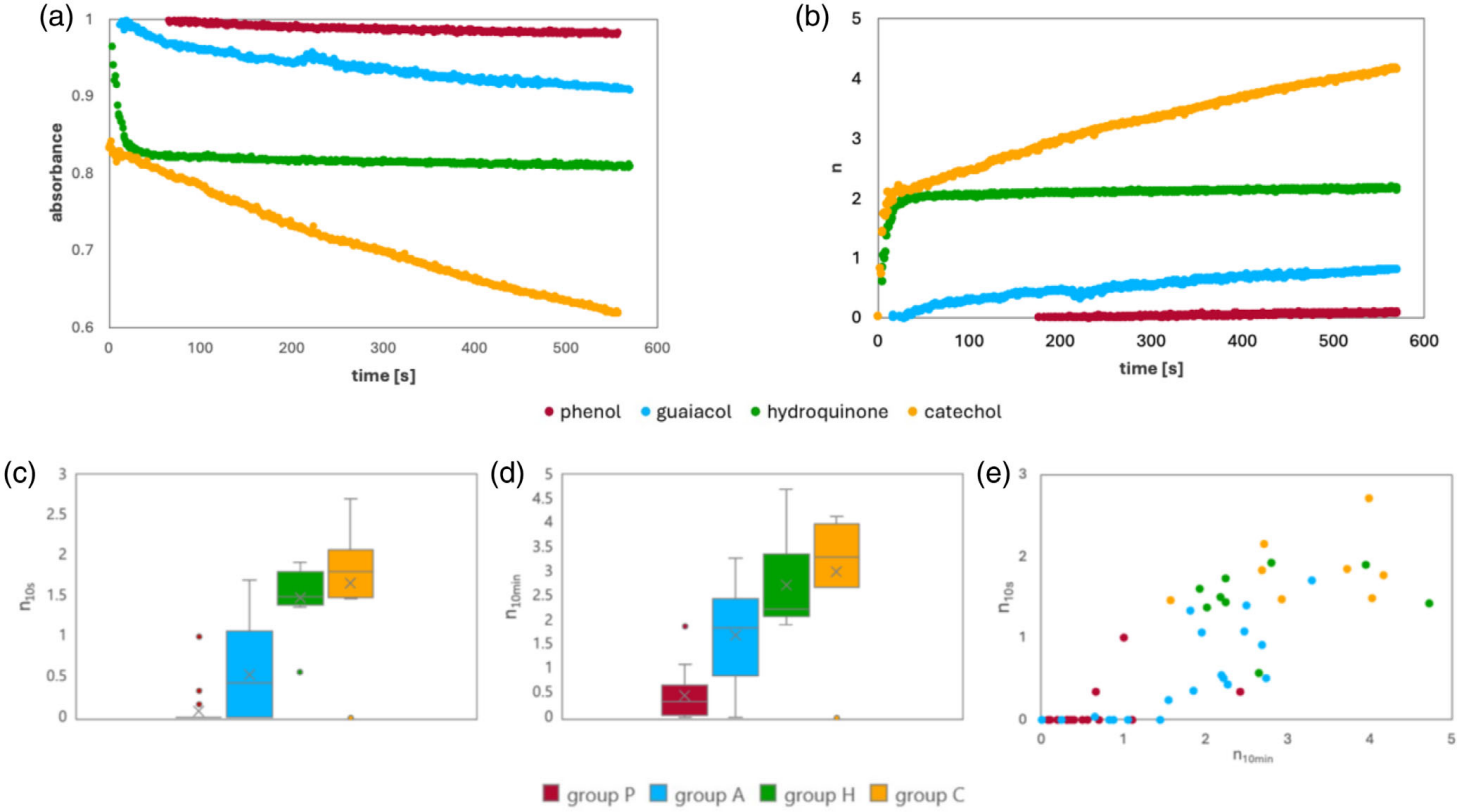
(a) Changes in absorbance as a function of time. (b) Changes in stoichiometric number (*n*) as a function of time. Box plots of *n* obtained (c) 10 s after the start of mixing (*n*_10s_) and (d) 10 min after the start of measurement (*n*_10min_). (e) Relationship between *n*_10min_ and *n*_10s_ for all investigated compounds. Values of *n*_10s_ and *n*_10min_ were measured under the condition of [DPPH]:[antioxidant] = 10:1, and values below 0 are presented as 0. Compounds with three hydroxy groups (gallic acid, pyrogallol and 1,2,4‐trihydroxybenzene) were excluded from this grouping.

The changes in the absorbance of phenol, methoxyphenol, hydroquinone and catechol, as well as *n* over time, are shown in Fig. 4(a),(b), respectively. In this study, compounds with methoxyphenol skeletons were classified as Group A, those with hydroquinone skeletons as Group H, those with catechol skeletons as Group C and those with other phenol skeletons as Group P. The distributions of *n*_10min_ and *n*_10s_ for each group are shown in Fig. 4(c)–(e). The *n*_10s_ of the groups increased in the order of Group P < Group A < < Groups C, H. The *n*_10min_ of Group P ranged from 0.042 to 0.61, that of Group A from 0.87 to 2.5, that of Group C from 2.1 to 3.4 and that of Group H from 2.7 to 4.0. The trend for *n*_10min_ was consistent with the results of our previous comprehensive measurement of 169 compounds (i.e. compounds with many hydroxyl groups have high TEAC values).^23^

Most of the compounds in Groups C and H undergo a fast reaction that scavenges two DPPH radicals within 10–20 s. For some compounds, this reaction is followed by a relatively slow reaction that scavenges approximately two more DPPH radicals. Most of the Group A compounds scavenge one or two DPPH radicals at a slower rate than compounds in Groups C and H.

#### Trends

**Figure 5:**
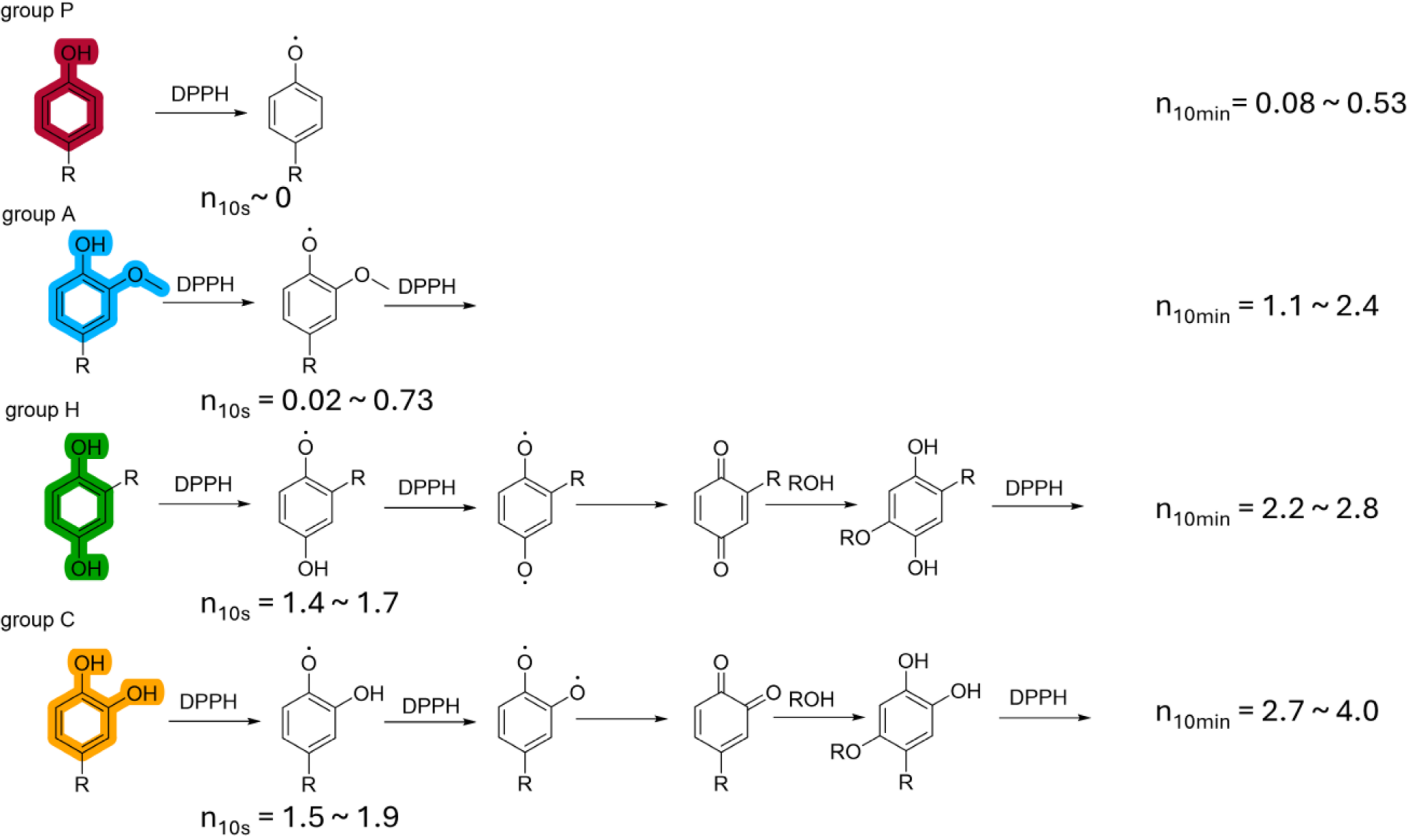
Reaction pathways of representative compounds for each basic skeleton. The stoichiometric numbers (*n*) obtained 10 min after the start of measurement (*n*_10min_) for the first and third quartiles are shown on the right. *n*_10s_ = *n* obtained 10 s after the start of mixing.

We discuss the trends observed for each group in relation to their possible mechanisms (Fig. 5, Table 1). The rapid scavenging of two DPPH radicals by catechol and hydroquinone corresponds to the reaction with two DPPH units to form a quinone, while the subsequent slow reaction occurs at the hydroxy group regenerated by alcohol attack and radical scavenging.^17^, ^18^ Groups C and H react faster than Group A because methoxy groups are weaker electron‐donating groups than hydroxy groups and the intramolecular hydrogen bond of the methoxy group inhibits the extraction of the hydrogen atom of the hydroxy group.^12^ One of the proposed reaction pathways for Group A compounds is the repeated reaction of DPPH radicals with the hydroxy groups regenerated upon dimerization.^25^, ^26^ Another pathway involves the withdrawal of hydrogen from methoxy groups by DPPH radicals.^25^, ^27^ In both reactions, one antioxidant unit reacts with two DPPH radicals. The ease of occurrence of each reaction is believed to determine the final *n*_10min_.

**Table 1:** Summary of radical scavenging activity, radical scavenging capacity and factors for each group

	Group P	Group A	Group H	Group C
Activity		↑	↑↑↑^a^	↑↑↑^a^
Explanation	Reaction rate is slower than those of Groups A, H and C	Phenolic hydroxy groups are stabilized by hydrogen bonding with methoxy groups, rendering the abstraction of hydrogen difficult	Hydroxy groups have strong electron‐donating properties, rendering them highly active	Hydroxy groups have strong electron‐donating properties, rendering them highly active. Generated radicals are stabilized by hydrogen bonding
Capacity		↑	↑↑	↑↑↑
Explanation	Reactions such as dimerization lead to an increase in capacity	Reactions such as dimerization and/or the removal of hydrogen from the position of the methoxy group lead to an increase in capacity	Reactions such as the regeneration of hydroquinone through the reaction of quinone and the solvent lead to increased reduction	Reactions such as the regeneration of catechol through the reaction of quinone and the solvent lead to increased reduction

^a^ In this study, the reaction rates of catechol and hydroquinone could not be compared because their reaction with two DPPH radicals was completed within a few seconds.

##### Substituent effects

**Figure 6:**
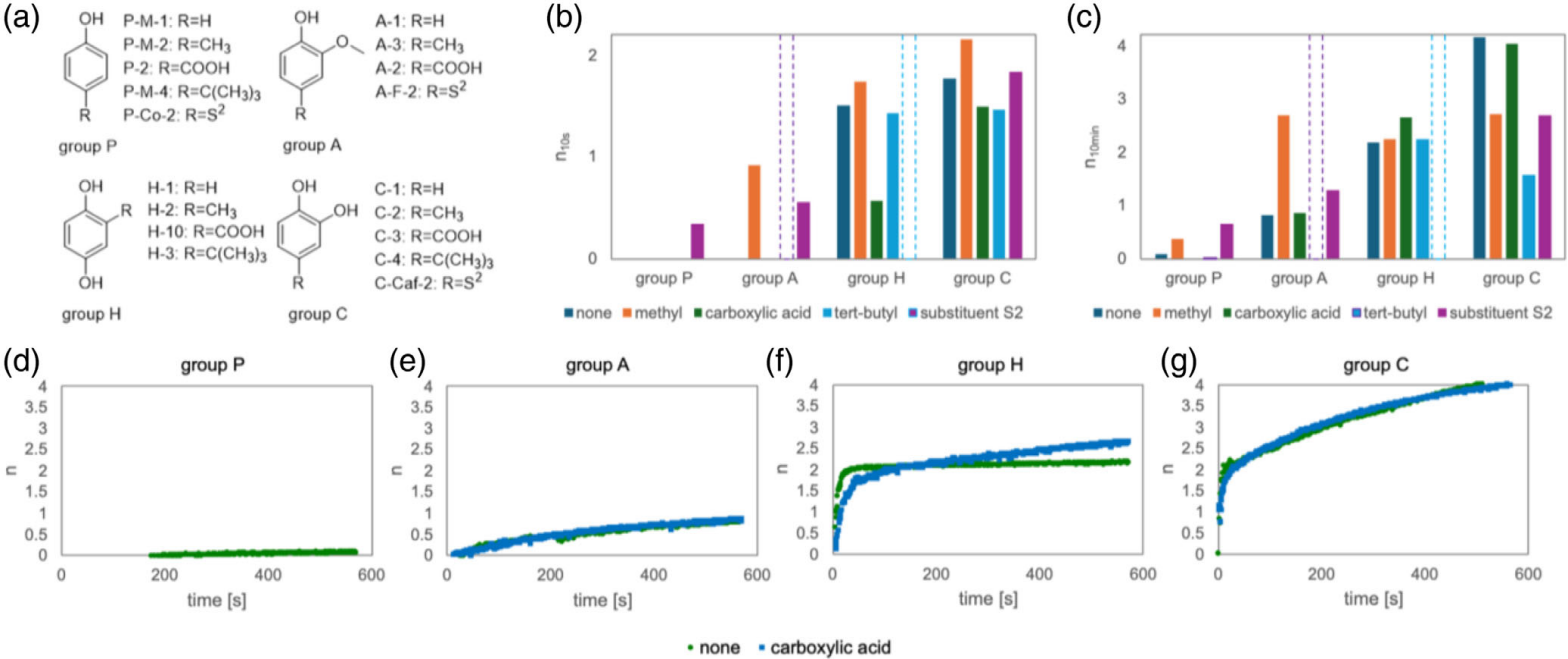
(a) General structures of the compounds in each group. Stoichiometric numbers (*n*) of phenol, methoxyphenol, hydroquinone, catechol and compounds with a methyl group or carboxylic acid obtained (b) 10 s after the start of mixing (*n*_10s_) and (c) 10 min after the start of measurement (*n*_10min_). Changes in *n* as a function of time for monomers and dimers of Groups (d) P, (e) A, (f) H and (g) C.

Bar charts for *n*_10s_ and *n*_10min_ are shown in Fig. 6(b),(c), respectively. Figure 6(d)–(f) shows the time evolution of *n* when carboxylic acids are introduced to groups with phenol, methoxyphenol, hydroquinone and catechol as their basic skeleton.

Table 2 summarizes the effects of various substituents on the reactions of each group. The introduction of carboxylic acid into phenol has been reported to increase its rate of reaction with DPPH and the amount of scavenged radicals, depending on the reaction conditions and substrate.^14^, ^17^ However, in all four groups investigated in this study, the introduction of carboxylic acid had no apparent effect on the reaction between the DPPH radicals and phenol (Fig. 6(d)–(g)) because the use of a buffer reduced the ionization ability and acidity of the carboxylic acid. When a *tert*‐butyl group serves as the electron‐donating group, an increase in activity is expected; however, the corresponding increase in steric hindrance leads to a decrease in activity and capacity. The balance between these effects is linked to an increase in *n*_10s_ and *n*_10min_ in each group. For example, the introduction of a single *tert*‐butyl group had no significant effect on *n*_10s_ and *n*_10min_ in Groups P and H but led to a decrease in *n*_10min_ compared with *n*_10s_ in Group C (*n*_10s_ was approximately 2; *n*_10min_ showed no change and was maintained approximately 2). Although no significant kinetic effect was observed following the introduction of the *tert*‐butyl group, alcohol attack and the subsequent regeneration of the hydroxy group may be impeded (Fig. 5).

**Table 2:** Effects of various substituents on *n*_10s_ ^a^ and *n*_10min_ ^b^

	—CH_2_R	—S^2^ (—C=CCOOH)	—H	—COOH	*tert*‐Butyl
Effect on *n*_10s_	↑	↑↑	Baseline	n.s.^c^	n.d.^d^
Explanation	Electron‐donating properties	Radical stabilization through conjugate extension	—	—	—
Effect on *n*_10min_	↑↑	↑	Baseline	n.s.^c^	↓↓ (Group C)
Explanation	Hydrogen abstraction at the benzyl position, etc.	Coupling at conjugated sites, etc.	—	—	Difficulty of alcohol addition to quinone caused by electron‐donating groups or steric hindrance (Group C)

^a^ Stoichiometric number obtained 10 s after the start of mixing.

^b^ Stoichiometric number obtained 10 min after the start of measurement.

^c^ n.s., not significant.

^d^ n.d., not determined.

Unlike *tert*‐butyl, the substitution group (—CH=CHCOOH) of the acrylic acid skeleton, which is conjugated, and electron‐donating groups with hydrogen in the adjacent position, such as methyl groups (—CH_3_) and the reduced form (—CH_2_CH_2_COOH) of acrylic acid and its derivatives, increased *n*_10s_ and *n*_10min_, as shown in the first and second columns of Table 2. Specifically, the introduction of a methyl group increased the activity (*n*_10s_) in the capacity (*n*_10min_); *n*_10min_ increased in Groups P, A and H, but decreased in Group C. The increase in *n*_10s_ is generally because methyl groups are electron donors and, hence, expected to increase the reaction rate. The increase in *n*_10min_ can be attributed to a reaction pathway in which the hydrogen at the benzyl position is extracted by the DPPH radical.^28^, ^29^ For example, among the compounds in Group A with one methoxy group, those with *n*_10min_ of 2 or greater (A‐3, A‐F‐3, A‐F‐7 and A‐M‐1) have a structure in which a hydrogen atom is present at the benzyl position. The occurrence of *n*_10min_ values larger than 2 may be attributed to the greater effects of hydrogen abstraction at the benzyl position. The decrease in *n*_10min_ in Group C is due to the nucleophilic addition of the alcohol to the quinone and inhibition of the accompanying regeneration of the hydroxy group by the electron‐donating methyl group.^17^ The increase in *n*_10s_ is due to the ability of the substitution group (—CH=CHCOOH) of the acrylic acid skeleton, which is believed to be an extension of the conjugated system, to stabilize phenoxy radicals.^30^, ^31^ Coupling at the conjugated site may also partly explain the increase in *n*_10min_. In this study, *n*_10min_ of A‐F‐2 was less than that of its reduced form, A‐F‐3, indicating that reduction leads to an increase in antioxidant capacity. However, *n*_10s_ of A‐F‐2 was greater than that of A‐F‐3. This increase in activity could be due to radical stabilization owing to the extension of the conjugated site. The increase in capacity could be attributed to the greater effect of hydrogen abstraction at the benzyl position than that of conjugated site coupling.

##### *O–o* coupling effects

**Figure 7:**
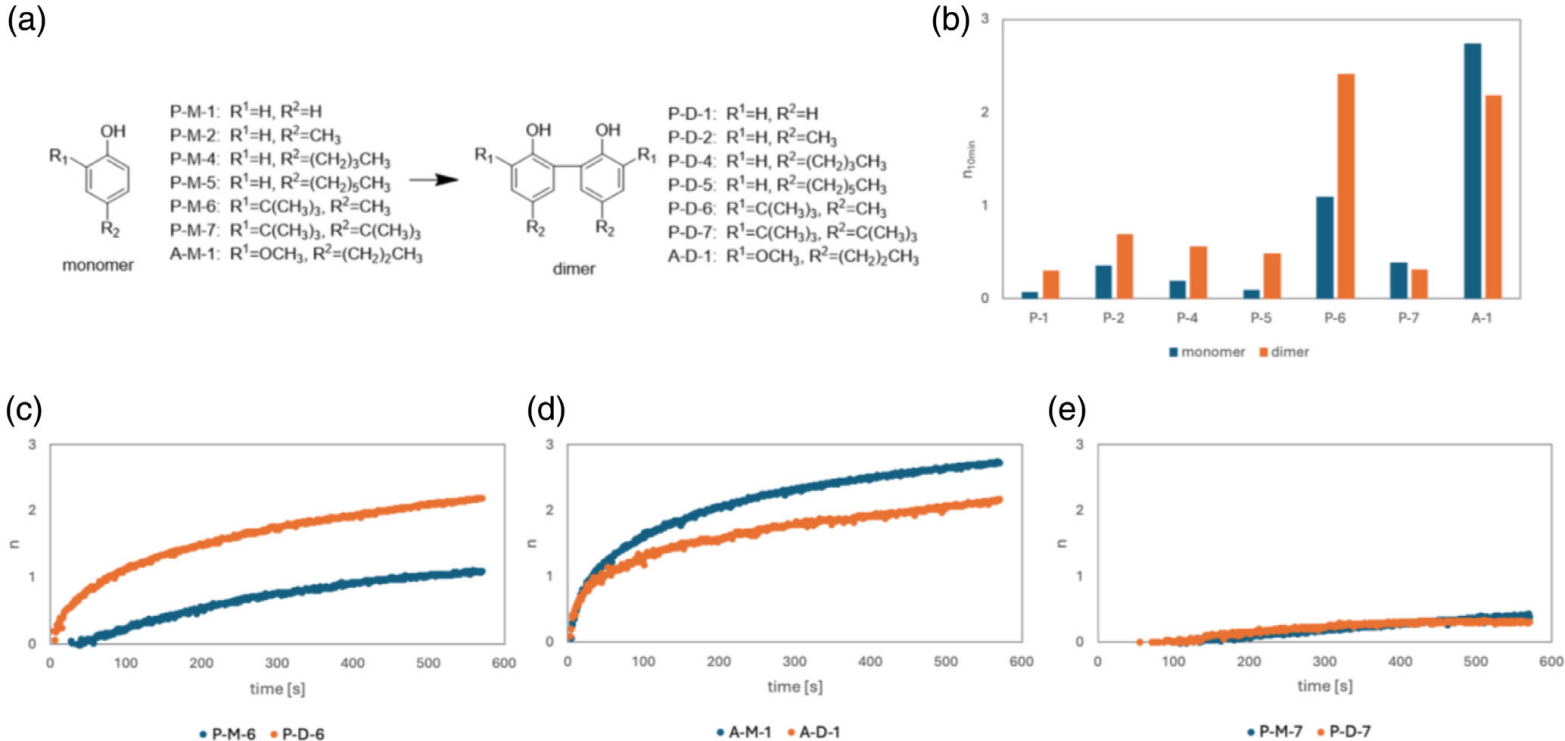
(a) Compound IDs and structures. (b) Comparison of the stoichiometric numbers (*n*) of phenol monomers and *o–o* dimers obtained 10 min after the start of measurement (*n*_10min_). Changes in *n* as a function of time for monomers and dimers of (c) P‐1, (d) A‐1 and (e) P‐7.

The RSA and RSC of the phenol monomer and *o–o* coupling dimer were evaluated. The structures of these compounds are shown in Fig. 7(a), and their calculated *n*_10min_ values are shown in Fig. 7(b). Although the effect of *o*‐*o* coupling was minor compared with the presence of catechol or a hydroquinone skeleton, a significant increase in *n*_10min_ could still be observed.

Most compounds showed an increase in *n*_10min_ owing to *o–o* coupling. As shown in Fig. 7(b), *n*_10min_ increased with increasing reaction rate for the dimers (P‐D‐1–6) compared with the monomers (P‐M‐1–6). This result may be attributed to the doubling of the number of hydroxy groups serving as reaction sites upon coupling and the decrease in BDE owing to the formation of intramolecular hydrogen bonds between adjacent hydroxy groups.^32^ Figure 7(e) shows a decrease in *n*_10min_ for A‐1, but no difference in *n*_10s_. The fact that A‐M‐1 shows *n*_10min_ close to 3 indicates the presence of a significant subsequent reaction. Dimerization at the *ortho* position is a potential subsequent reaction of phenoxy radicals after the reaction of phenol with DPPH.^25^, ^26^ The decrease in *n*_10min_ may be due to changes in the subsequent reactions associated with dimerization. Unlike the other compounds, P‐6 (Fig. 7(e)) showed no increase in *n*_10s_ and *n*_10min_ because it has a *tert‐*butyl group at the *ortho* and *para* positions, resulting in a highly sterically hindered structure that becomes bulkier upon dimerization. Therefore, the effects of increased reaction rates owing to coupling and intramolecular hydrogen bonding and decreased reaction rates owing to the increase in steric hindrance may cancel each other out, resulting in no observable change in reaction rate.

## CONCLUSION

This study systematically evaluates the kinetic and thermodynamic aspects of DPPH radical scavenging by 56 phenolic compounds commonly found in food, forming the largest dataset of its kind for parallel analysis of RSA and RSC. By introducing time‐resolved measurements (*n*_₁₀s_ and *n*_₁₀min_), we demonstrated that traditional antioxidant indices such as TEAC primarily reflect the thermodynamic endpoint (*n*_₁₀min_), whereas kinetic behavior (*n*_₁₀s_) provides crucial complementary insights into antioxidant activity. Group‐wise comparisons based on structural similarity revealed distinct reactivity patterns among compound classes and clarified the divergent roles of specific substituents. Our findings reaffirm the well‐known high activity of catechol and hydroquinone structures while further elucidating how specific structural variations – including methoxy substitution, conjugation, steric hindrance and *o–o* coupling – affect both RSA and RSC. Notably, we identified structural motifs (e.g. benzyl‐position hydrogens, extended conjugation and dimerization) that enable certain compounds to exceed the expected stoichiometric number of two, suggesting additional mechanisms such as hydrogen abstraction and regeneration pathways. This integrated kinetic–thermodynamic evaluation provides a more nuanced understanding of antioxidant behavior and offers a rational basis for designing or selecting phenolic antioxidants based on structure–activity relationships. Ongoing efforts focus on combining this experimental dataset with machine learning approaches to develop generalized predictive models for antioxidant performance.^33^, ^34^, ^35^, ^36^

## Supporting information


Data S1.

Data S2.

## Data Availability

The data that supports the findings of this study are available in the supplementary material of this article.
