# Supplementary material for: Kinetic and thermodynamic evaluation of antioxidant reactions: factors influencing the radical scavenging properties of phenolic compounds in foods
Source: J Sci Food Agric. 2025 Jul 24;105(14):8186–95. doi: 10.1002/jsfa.70080 (PMC12509046; doi:10.1002/jsfa.70080)
Supplement: Supplementary file 2 — Data S2. [file JSFA-105-8186-s002.docx]

Kinetic and Thermodynamic Evaluation of Antioxidant Reactions: Factors Influencing the Radical Scavenging Properties of Phenolic Compounds in Foods

**Running Title:** Considerations for the antioxidant properties of phenolic compounds

**Moeka Yamauchi, Yukino Kitamura, Chihiro Tada, Riku Kato, and Hiroaki Gotoh***

# Attached File

An Excel file is attached with supplementary information on the measurement results, including the names of the compounds measured, n10min, n10sec and their standard deviations, TEAC values and references.

- measured_values.xlsx


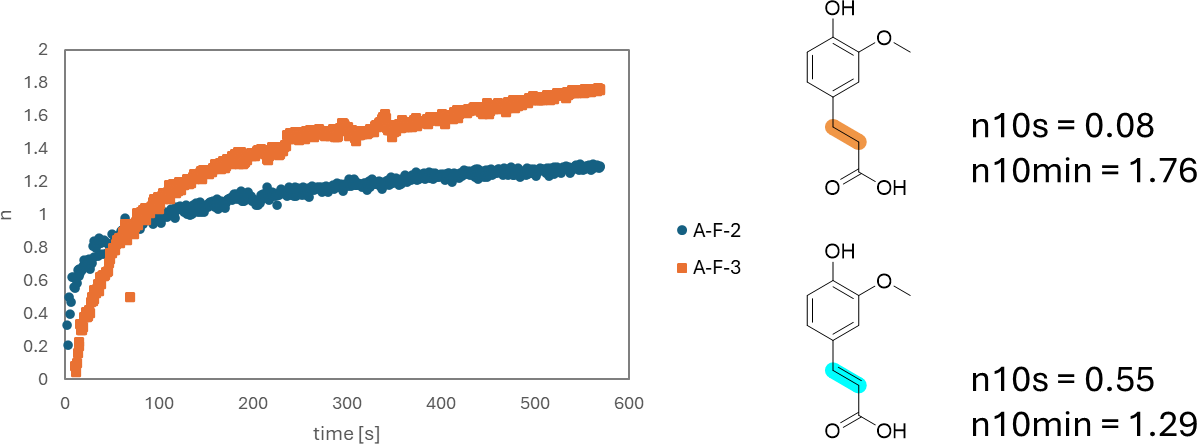
The change in n over time (10 min) before and after the reduction of ferulic acid is shown in Figure SI-1.

Figure SI-1 n versus time of sinapic acid and dihydrosinapic acid.

# Synthesis Procedure


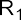

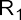

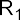

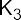


The following procedure was used to synthesize **P-D-3**: 0.9 mmol of the raw material and


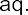


1.8 mL of pure water were put into the reaction vessel and stirring was started. Anhydrous iron(III) chloride 0.6 mmol was added directly to the system and allowed to react for 24 hours at room temperature. After the reaction, the organic layer was extracted with chloroform. The organic layer was dehydrated with sodium sulfate and the solvent was removed under reduced pressure to give the crude product. Isolation was performed by thin layer chromatography (SiO2, Ethyl acetate:Hexane) to obtain **P-D-3**. Following this procedure, we also carried out the synthesis of P-D-6 and P-D-8. The NMR spectra of the compounds are shown below.

## P-D-6

**
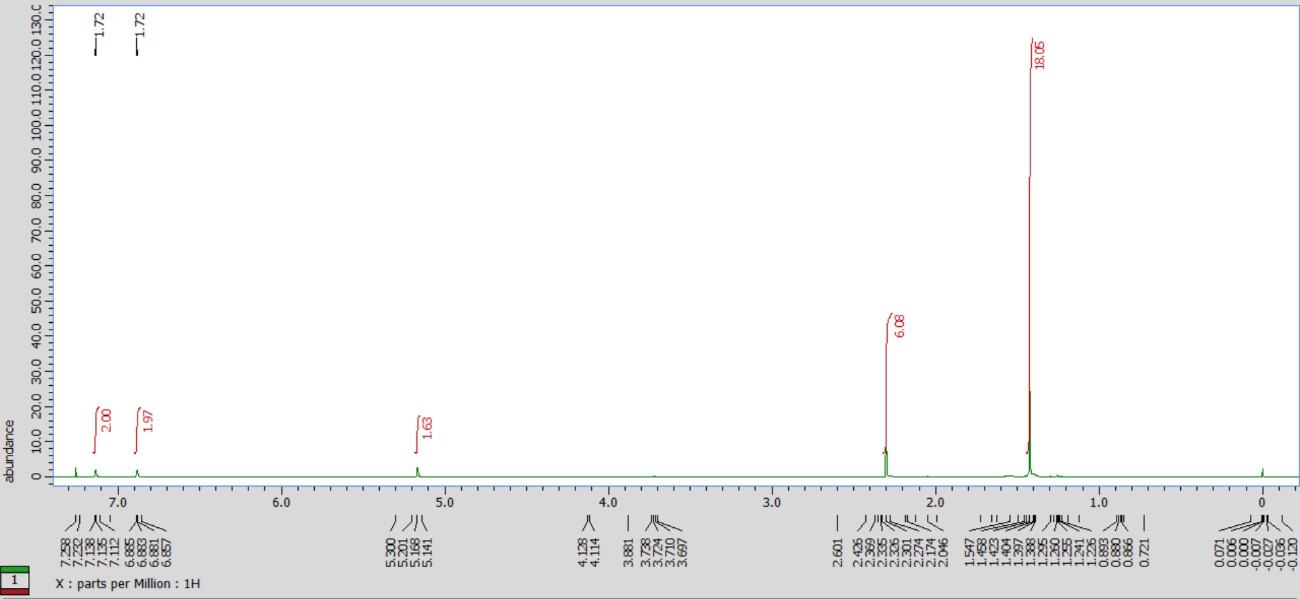
**

1H-NMR (500 MHz, CHLOROFORM-D) δ 7.14 (d, *J* = 1.7 Hz, 2H), 6.88 (d, *J* = 1.7 Hz, 2H), 2.30 (s,

6H), 1.42 (s, 18H)

## P-D-7

**
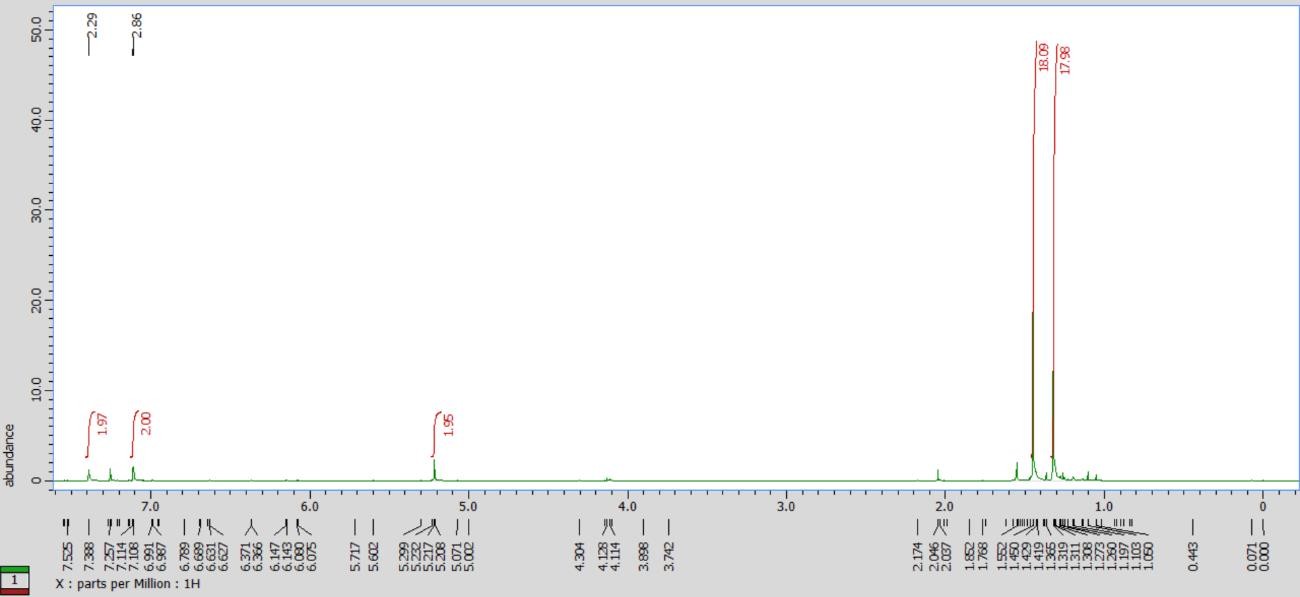
**

1H-NMR (500 MHz, CHLOROFORM-D) δ 7.39 (d, J = 2.3 Hz, 2H), 7.11 (d, J = 2.9 Hz, 2H), 1.45 (s,

18H), 1.32 (s, 18H)
